# Network pharmacological approach combined with weighted gene co-expression network analysis identifies CDKN2A as the keg target of Changweiqing against colorectal cancer

**DOI:** 10.1186/s41065-025-00405-8

**Published:** 2025-03-10

**Authors:** Ma Zushuai, Ji Yanrong, Zhao Chengdu, Zhu Xu, Ding Qianshan

**Affiliations:** 1 Department of Gastroenterology, Renmin Hospital of Wuhan University, Zhongxiang, Hubei 431900 China; 2 Department of Gastroenterology, Zhongxiang People’s Hospital, Zhongxiang, Hubei 431900 China; 3 Department of Gastrointestinal Surgery, Renmin Hospital of Wuhan University, Wuhan, Hubei 430060 China

**Keywords:** Changweiqing, Colorectal cancer, CDKN2A, Network pharmacology, WGCNA

## Abstract

**Background and objective:**

Changweiqing (CWQ) is a Chinese herbal formula for the treatment of the gastrointestinal tract diseases, but its role in the treatment of colorectal cancer (CRC) has not been clarified. This study aimed to explore the molecular mechanism of CWQ in CRC treatment through bioinformatics analysis and network pharmacology.

**Methods:**

Traditional Chinese Medicine Systems Pharmacology Database and Analysis Platform and SwissTargetPrediction database were used to collect the bioactive components of CWQ. The databases including DisgeNET, GeneCards, MalaCards, Online Mendelian Inheritance in Man and Comparative Toxicogenomics were used to obtain CRC-related targets. The Cancer Genome Atlas - colon adenocarcinoma dataset was used to obtain prognosis-related genes in CRC based on weighted gene co-expression network analysis (WGCNA). A protein-protein interaction network was constructed to screen core targets, with STRING database and Cytoscape software. Gene Ontology and Kyoto Encyclopedia of Genes and Genomes enrichment analyses were performed using the Database for Annotation, Visualization and Integrated Discovery database. Molecular docking was performed with AutoDock Vina software. Core targets were further analyzed using Gene Expression Profiling Interactive Analysis platform, Human Protein Atlas database, University of ALabama at Birmingham CANcer data analysis Portal (UALCAN) and GeneMANIA database. In vitro experiments were further performed to investigate the effects of quercetin, one of the main components of CWQ, on CRC cells.

**Results:**

6356, 1901 and 2980 CRC-related genes were obtained from differential expression analysis, WGCNA and open access databases, respectively. CWQ contained a total of 70 bioactive ingredients, of which 64 ingredients had a total of 836 therapeutic targets. Functional enrichment analysis indicated that CWQ may be involved in regulating pathways in cancer, MAPK signaling pathway and AGE-RAGE signaling pathway, and further analysis identified 14 core targets of CWQ. These core targets were significantly correlated with cell cycle, p53 signaling pathway, FoxO signaling pathway and pathways in cancer. Among these core targets, cyclin-dependent kinase inhibitor 2 A (CDKN2A) expression was closely associated with shorter overall survival and clinical stage of CRC patients. The main bioactive ingredients of CWQ targeting CDKN2A were quercetin, luteolin, kaempferol, isorhamnetin, 7-O-methylisomucronulatol and 7-Methoxy-2-methyl isoflavone. Additionally, quercetin caused G0/G1 phase arrest and inhibited the viability of CRC cells.

**Conclusion:**

The active ingredients of CWQ may play an anti-CRC role through multi-targets and multi-pathways, regulating the cell cycle and cell viability of CRC cells.

**Supplementary Information:**

The online version contains supplementary material available at 10.1186/s41065-025-00405-8.

## Introduction

Colorectal cancer (CRC) is one of the most common gastrointestinal malignancies, with approximately 2 million patients diagnosed each year [1, 2]. Incidence rates increased during 2015–2019 by 1-2% annually for CRC [3, 4]. In recent decades, a variety of emerging therapeutic strategies have been used to treat human malignancies, including targeted therapy and immune checkpoint inhibitor therapy [3, 4]. CRC exhibits strong heterogeneity and metastatic potential, which makes clinical treatment still face great challenges [5]. Therefore, there is an urgent need for new, less toxic and effective drugs for its treatment.

Traditional Chinese medicine is currently playing an important role in cancer treatment as a complementary treatment and alternative strategy [6]. Natural drugs often have multi-target effects and can regulate multiple biological pathways, so as to achieve the purpose of treating and preventing diseases. This multi-target action not only improves the effectiveness of the treatment, but also helps reduce the side effects of the drug. In addition, the many bioactive ingredients in natural medicines often have synergistic effects and can enhance or antagonize each other, further improving the efficacy and safety of the drug. Because natural drugs are derived from nature, their components are relatively complex but most of them are biocompatible [7–11]. Many natural compounds present in herbs have anti-tumor properties [6, 12]. For example, esculin, a bioactive compound derived from *cortex fraxini*, promotes CRC cell apoptosis by inducing ferroptosis [13]. Emodin is a natural anthraquinone derivative in many widely used Chinese medicinal herbs, which can inhibit proliferation and induced apoptosis in CRC cells [14]. Changweiqing (CWQ) is a widely used Chinese herbal medicine formula, which mainly includes *Hedysarum Multijugum Maxim*, *Codonopsis Radix*,* Atractylodes Macrocephala Koidz.*, *Polyporus Umbellatus(Pers)Fr.*, *Coicis Semen*, *Sargentodoxae Caulis*, *Akebia quinata (Thunb.) Decne* and *Vitis quinquangularis Rehder* [15]. It is reported that, in cell model, CWQ can repress tumor growth and aggressiveness of CRC, and reverse vincristine resistance [15–18]. In addition, CWQ shows the potential to inhibit the tumorigenesis of CRC by improving intestinal barrier function [19]. CWQ treatment can also alleviate oxaliplatin-triggered side effects and reverse platinum drug resistance [20]. However, the molecular mechanism of CWQ in CRC treatment has not been fully elucidated, which blocks its further application in clinical practice.

Multiple omics such as transcriptomics, proteomics, and metabolomics have been developed to identify novel biomarkers of various diseases, including CRC [21]. Weighted gene co-expression network analysis (WGCNA) is a widely used algorithm for gene co-expression network construction [22]. WGCNA is helpful to find gene modules of highly related genes and correlate them with certain features (such as various clinical features) [23, 24]. Network pharmacology is widely used to reveal the components and drug targets of herbal medicines [25, 26]. In this study, WGCNA was used to identify the genes associated with CRC progression, and network pharmacology and molecular docking were combined to explore the targets of CWQ in CRC treatment.

## Materials and methods

### Data processing

From UCSC database (https://xenabrowser.net/datapages/), HTSeq-Counts, HTSeq-FPKM and phenotype data of The Cancer Genome Atlas Program (TCGA) - Colon adenocarcinoma (COAD) cohort were downloaded. The gene expression profiles included the data of 41 non-cancer samples and 471 cancer samples. The phenotype file included clinical information for 569 patients. To identify the differentially expressed genes (DEGs), HTSeq-Counts data and R package DESeq2 were used. The threshold was set to |log_2_fold change| >| 2.0 and padj < 0.05. ggplot2 and pheatmap packages in R software (version 4.3.1, 2023.11.17) were applied to draw the volcano map and heatmap of DEGs. With HTSeq-FPKM data, R package WGCNA was used to construct gene co-expression network [27]. The soft threshold power β was 6, and cutHeight = 0.6, and minModuleSize = 20. The gene significance (GS) and module membership (MM) were calculated. A correlation coefficient > 0.3 and *P* < 0.05 were used to obtain the key modules.

### Acquisition of CRC-related genes from open access databases

Using DisgeNET datbase (https://www.disgenet.org/), GeneCards database (https://www.genecards.org/), MalaCards database (https://www.malacards.org/), Online Mendelian Inheritance in Man (OMIM) database (https://omim.org/about) and Comparative Toxicogenomics Database (CTD, http://ctdbase.org/), the genes related with CRC tumorigenesis and progression were retrieved. All duplicate genes were deleted to obtain CRC-related targets.

### Active ingredients and targets of CWQ

The active ingredients of CWQ were collected by Traditional Chinese Medicine Systems Pharmacology Database and Analysis Platform (TCMSP, http://lsp.nwu.edu.cn/) and HERB database (http://herb.ac.cn/). The screening conditions were oral bioavailability (OB) ≥ 30% and drug-likeness (DL) ≥ 0.18. OB ≥ 30% have high oral absorption and slow metabolism [28]. DL ≥ 0.18 have better effects in drug development.

From TCMSP and PubChem database (https://pubchem.ncbi.nlm.nih.gov/), canonical SMILES of the active ingredients was obtained. With SwissTargetPrediction platform (http://www.swisstargetprediction.ch/), the targets of the active ingredients were obtained. The targets of all active ingredients were collected, and the drug targets of CWQ were obtained after the duplicates were deleted. The drug-ingredient-target network diagram was constructed using Cytoscape software (version 3.9.1, 2023.10.1).

### Gene ontology (GO) and Kyoto encyclopedia of genes and genomes (KEGG) analyses

Using the Database for Annotation, Visualization, and Integrated Discovery (DAVID, version 6.8) (http://david.abcc.ncifcrf.gov/) [27, 28], functional annotations of the target genes was performed with GO analysis and KEGG analysis. The significant items were defined as the gene sets with *P* < 0.05 and containing more than 5 genes. GO analyses, including biological processes (BP), cell components (CC), and molecular function (MF), were used to predict the biological function of the genes.

### Cyclin-dependent kinase inhibitor 2 A (CDKN2A) expression and characteristic analysis

The relationship between gene expression and overall survival (OS) and clinical stage of CRC patients was analyzed using the Gene Expression Profiling Interactive Analysis (GEPIA) database (http://gepia2.cancer-pku.cn/#index). The University of ALabama at Birmingham CANcer data analysis Portal (UALCAN) (https://ualcan.path.uab.edu/index.html) was used to analyze gene methylation level. Human Protein Atlas (HPA) database (https://www.proteinatlas.org/) was used to show protein subcellular localization. Functionally similar proteins of target proteins were analyzed in the GeneMANIA (http://genemania.org/) database.

### Molecular docking

The 3D crystal structure of CDKN2A (PDB: 1BI7) was obtained from the Structural Bioinformatics Research Collaboration (RCSB) Protein database (PDB, https://www.RCSB.org/). 3D structure of small molecules was obtained from PubChem database. CDKN2A and small molecule structures were processed with AutoDock tools, including removal of bound ligands and water molecules, calculation of Gasteiger charges, addition of polar hydrogen and combination of non-polar hydrogen. CDKN2A was set as the receptor and the small molecule was set as the ligand. Molecular docking was then performed via AutoDock Vina (version 1.5.7, 2023.11.24). Finally, the receptor-ligand complex was imported into Pymol software (version 2.4.0, 2023.11.24) and Ligplus software (version 2.2.4, 2023.11.24) to analyze the binding pattern between the receptor and ligand.

### Cell culture

Human colonic cancer cell lines HT-29 and SW-480 were purchased from Beyotime (Shanghai, China). The cells were cultured in RPMI-1640 medium containing 10% fetal bovine serum, 100 U/mL penicillin, and 0.1 mg/mL streptomycin, at 37℃ with 5% CO_2_, in a humidified incubator.

### Cell viability assay

CRC cell proliferation was detected by a Cell Counting Kit-8 (CCK-8) Assay kit (Beyotime, Shanghai, China). In brief, CRC cells were inoculated in 96-well plates at an initial density of 1 × 10^3^ cells/well and cultured in media without or containing quercetin (concentrations of 0, 50, 100, 150, 200, or 250 µM) for 24–48 h. Then 10 µL CCK-8 reagent was added to the well and the cell culture was continued for 2 h. After that, using an ultra-microplate reader (EMax; Molecular Devices, USA), the optical density of each well at 450 nm wavelength was detected, and the data were analyzed to calculate IC_50_.

### Analysis of cell cycle

CRC cells were inoculated in a 60 mm petri dish with a density of 5 × 10^5^ cells. This was followed by treatment with specified doses of quercetin (100 and 200 µM) for 48 h. The cells were collected using trypsin, and then washed with phosphate-buffered saline (PBS). The cells were collected and fixed overnight with 75% ethanol at -4 ° C. On the second day, the cells were washed with PBS and incubated with 100 µl RNase A at 37℃ for 30 min, and then stained with 400 µl propyl iodide (PI) in the dark for 30 min. Finally, a FACScan flow cytometer (BD Biosciences, CA, USA) was used to measure cell cycle distribution.

### Reverse transcription-quantitative polymerase chain reaction (RT-qPCR)

CRC cells were collected and TRIzol reagent (Invitrogen, Shanghai, China) was used to extract total RNA. Total RNA was then reverse-transcribed into cDNA using the PrimeScript™ RT reagent Kit (Perfect Real Time) (Takara, Dalian, China). RT-qPCR was performed using a SYBR Premix Ex Taq Kit (Takara, Dalian, China) in CFX96 Touch Real-Time PCR Detection system (Bio-Rad Laboratories, Hercules, CA, USA). The 2^−ΔΔCt^ method was employed to quantify gene expression, and relative mRNA expression levels were normalized to mRNA levels of glyceraldehyde-3-phosphate dehydrogenase (GAPDH). All primers were synthesized by GenePharma Co., Ltd. (Shanghai, China). The priming sequences are as follows: cyclin D1 (CCND1) forward primer 5 ‘-GTCGCTGGAGCCCGTGAA-3’ and reverse primer 5’ -GGCCGGATGGAGTTGTCG-3’. Cyclin E1 (CCNE1) forward primer 5’-TACACCAGCCACCTCCAGAC-3’ and reverse primer 5’-CCTCCACAGCTTCAAGCTTTTG-3’. Cyclin dependent kinase 2 (CDK2) forward primer 5’-CGCAAATGCTGCACTACGACC-3’ and reverse primer 5’-GCCCACCTGAGTCCAAATAGCC-3’. Cyclin dependent kinase 4 (CDK4) forward primer 5’-GGGGACCTAGAGCAACTTACT-3’ and reverse primer 5’-CAGCGCAGTCCTTCCAAAT-3’. p21 forward primer 5’-CTGGAGACTCTCAGGGTCGA-3’ and reverse primer 5’-CGGATTAGGGCTTCCTCTTG-3’. GAPDH forward primer 5’-TGGCACCCAGCACAATGAA-3’ and reverse primer 5’-CTAAGTCATAGTCCGCCTAGA-3’.

### Statistical analysis

The data was analyzed using GraphPad Prism software (version 7.00, 2023.12.29) and shown as “mean ± standard deviation”. All experiments were repeated at least 3 times. The one-way analysis of variance (ANOVA), followed by Tukey *post-hoc* test, was used for statistical analysis. A *P* value of less than 0.05 indicated that the results were statistically significant.

## Results

### Identification of CRC-related genes

**Fig. 1:**
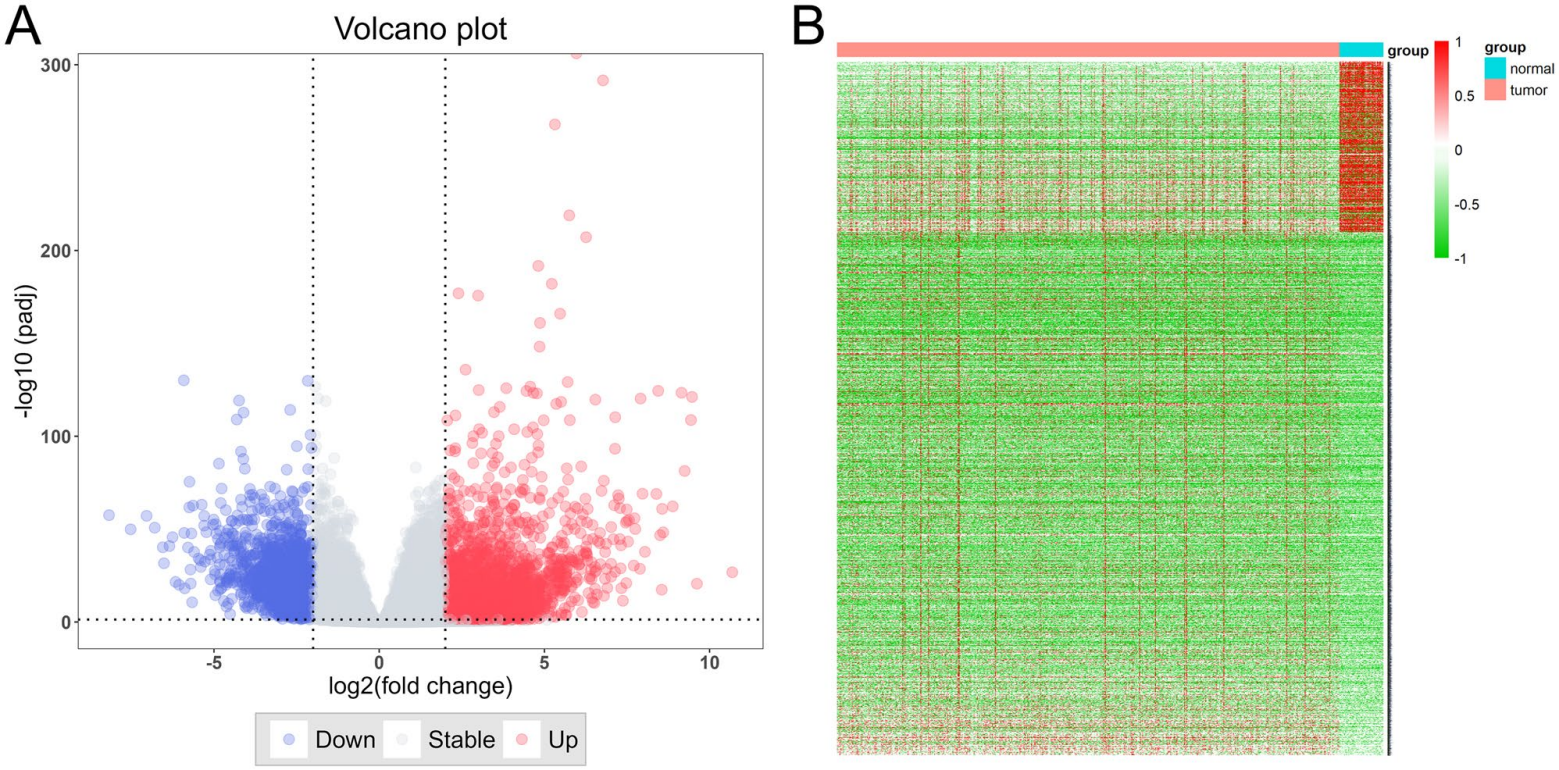
DEGs of CRC tissues in TCGA-COAD cohort. (**A**) The volcano map showing DEGs between cancer and non-cancer samples in TCGA-COAD cohort. Blue represents down-regulated genes and red represents up-regulated genes. (**B**) The heat map shows the expression profile of DEGs in the TCGA-COAD cohort

**Fig. 2:**
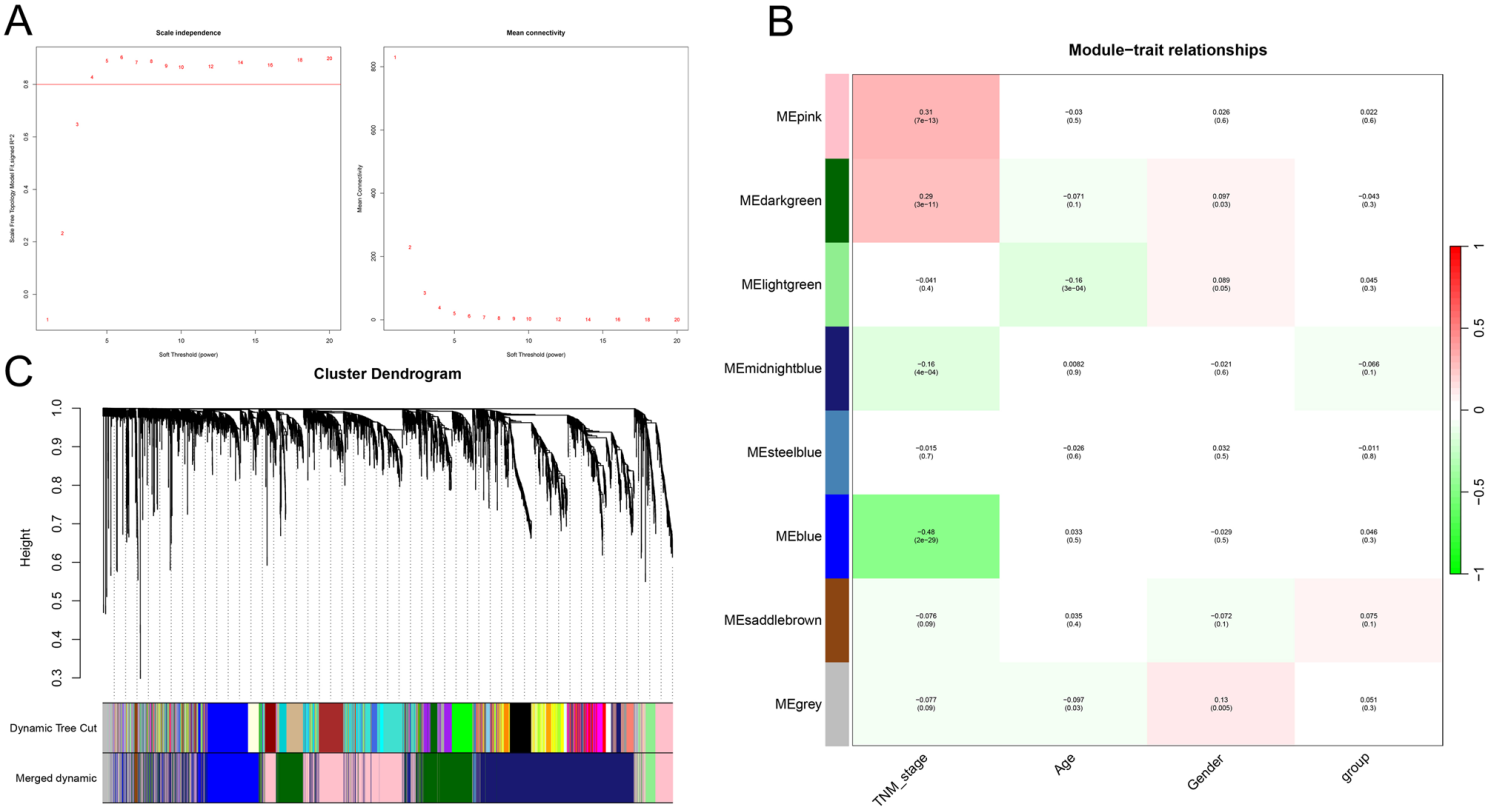
The genes associated with clinical features of CRC are identified by WGCNA. (**A**) Scale independence and mean connectivity in WGCNA. (**B**) Gene dendrogram and modules in WGCNA. (**C**) Pearson correlation analysis between modules and clinical features of CRC patients

Through differential expression analysis of the TCGA-COAD dataset, 6356 DEGs were obtained, among which 4789 were up-regulated genes and 1567 were down-regulated genes (Fig. 1A&B). Next, 569 samples and 5000 genes were analyzed by WGCNA, and no outliers were detected in the hierarchical clustering of samples. The soft threshold was set to 6, and the gene co-expression network was constructed according to the scale-free criteria (Fig. 2A). Subsequently, eight modules were identified, namely blue, grey, midnightblue, pink, lightgreen, saddlebrown, darkgreen, and steelblue modules (Fig. 2B). TNM stage was strongly correlated with blue module (Cor=-0.48, *P* = 2e-29) and pink module (Cor = 0.91, *P* = 7e-13) (Fig. 2C). So, the genes in the blue and pink modules, 1901 genes, were considered to be associated with CRC tumorigenesis and progression. Additionally, from DisgeNET (C0009402, Inference score > 50), GeneCards (Relevance score > 13.5), MalaCards, OMIM and CTD (Inference Score > 50) databases, 1114, 1178, 917, 175 and 1740 CRC-related genes were obtained, respectively. Collectively, 2980 CRC-related genes were obtained after removing the duplicates, from the open access databases.

### Identification of drug targets of CWQ

**Fig. 3:**
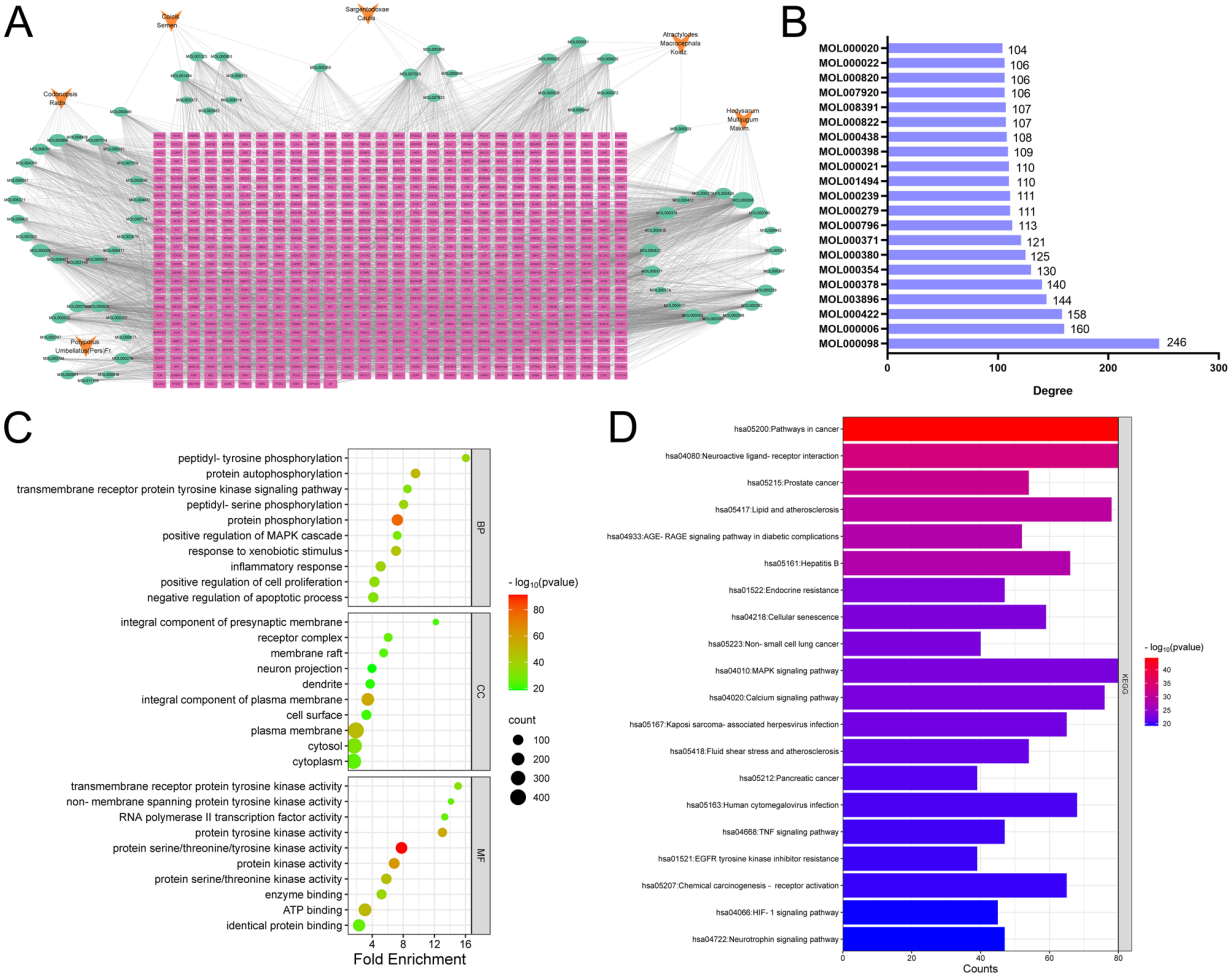
The drug-ingredient-target network of CWQ and cluster analysis. (**A**) Network diagram of CWQ active ingredients and targets. Orange nodes represent the drug, green nodes represent the active ingredient, and pink nodes represent the target. (**B**) The bar chart showing the degree values of the active ingredients in CWQ (> 100). (**C**) The bubble map showing the GO analysis results of CWQ’s targets (top 10), including biological processes (BP), cellular components (CC) and molecular function (MF). (**D**) The bar chart showing the results of KEGG enrichment analysis of CWQ’s targets (top 20)

Through TCMSP and HERB databases, a total of 70 active ingredients were obtained (Table S1). Only 64 of these components were predicted to have targets, and a total of 836 targets were obtained. The drug-ingredient-target network was constructed using Cytoscape, which included 899 nodes (6 herbs, 70 components, and 823 genes), 4322 edges (Fig. 3A). Of these, 21 active ingredients had a degree value greater than 100, including quercetin (MOL000098, degree = 246), luteolin (MOL000006, degree = 160), kaempferol (MOL000422, degree = 158), 7-Methoxy-2-methyl isoflavone (MOL003896, degree = 144), 7-O-methylisomucronulatol (MOL000378, degree = 140), isorhamnetin (MOL000354, degree = 130), (6aR,11aR)-9,10-dimethoxy-6a,11a-dihydro-6 H-benzofurano[3,2-c]chromen-3-ol (MOL000380, degree = 125), 3, 9-di-o-methylnissolin (MOL000371, degree = 121), (22e,24r)-ergosta-6-en-3beta,5alpha,6beta-triol (MOL000796, degree = 121) degree = 113), Cerevisterol (MOL000279, degree = 111) (Fig. 3B). GO analysis showed that the drug targets were related to biological processes including protein phosphorylation, response to xenobiotic stimulus and inflammatory response; related to cellular components including plasma membrane, cytosol and receptor complex; related to molecular functions such as protein kinase activity, ATP binding and enzyme binding (Fig. 3C). KEGG enrichment analysis showed that drug targets were associated with pathways in cancer, neuroactive ligand-receptor interaction, lipid and atherosclerosis, AGE-RAGE signaling pathway in diabetic, cellular senescence, MAPK signaling pathway, TNF signaling pathway, and HIF-1 signaling pathway (Fig. 3D).

### Screening core targets of CWQ in CRC treatment

**Fig. 4:**
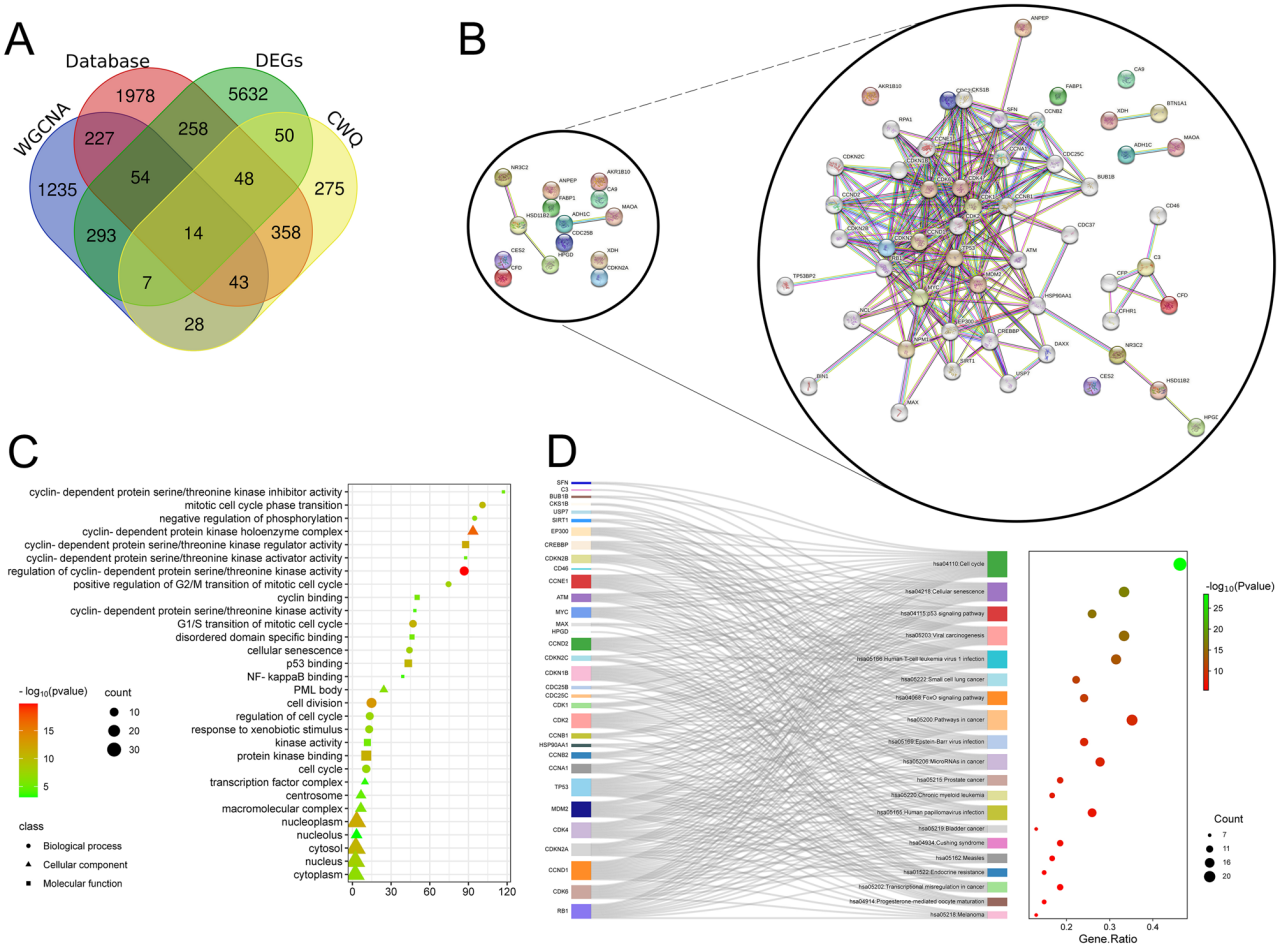
Core target screening and cluster analysis of CWQ’s targets in CRC treatment. (**A**) The venn diagram shows the common genes of the CRC-related genes from DEGs, WGCNA, open databases and CWQ targets. (**B**) The PPI network of 14 identified common genes was constructed with STRING database. (**C**) The bubble map showing the GO analysis results of 54 genes (top 10), including biological processes (BP), cellular components (CC) and molecular function (MF). (**D**) The Sankey diagram showing the results of KEGG enrichment analysis of 54 genes (top 20)

The CRC-related genes retrieved from DEGs, WGCNA and open access databases were combined with CWQ targets, and the Venn diagram showed that there were 14 common genes, including XDH, CDC25B, MAOA, AKR1B10, HSD11B2, CES2, ADH1C, CFD, HPGD, FABP1, ANPEP, CA9, CDKN2A, NR3C2 (Fig. 4A). Subsequently, protein-protein interaction (PPI) network was constructed, and protein interaction analysis was performed through the STRING database (https://cn.string-db.org/), and the minimum required interaction score was 0.700 (high confidence), and a network was obtained, which included 54 nodes (that indicated that there were 54 genes in the PPI network) and 252 edges, with an average node degree of 9.33 (Fig. 4B). The degree, betweenness and closeness values were calculated by topological analysis using CytoNCA plug-in in Cytoscape software. CDKN2A had the highest degree value among the 14 genes (degree = 19, betweenness = 19.2, closeness = 0.09); the second was cell division cycle 25B (CDC25B) (degree = 9, Betweenness = 1.7, Closeness = 0.09) (Table 1). Among these genes, AKR1B10, CA9, CES2 and FABP1 were independent proteins in the network, that were not included in the calculation (Fig. 4B). In addition, cluster analysis was carried out by MCODE plug-in on Cytoscape software. Notably, the results showed that the network cluster consisted of 2 modules with scores of 11.167 and 4.909 respectively, and only CDKN2A and CDC25B, among the 14 genes, were in module 1 and module 2 respectively (Figure S1). GO analysis showed that, for BP, these 54 genes were related to cell cycle, division and senescence; for CC, these genes were related to cyclin-dependent protein kinase holoenzyme complex, nucleoplasm and cytosol; for MF, these genes were related to combination with cyclin-dependent protein serine/threonine kinase regulator activity, protein kinase binding and p53 binding (Fig. 4C). KEGG enrichment analysis showed that these genes were correlated with cell cycle, cellular senescence, p53 signaling pathway, FoxO signaling pathway and pathways in cancer (Fig. 4D).

**Table 1:** Topological analysis of the genes in PPI network

	Gene name	Degree	Betweenness	Closeness
1	TP53	29	255.6158	0.097222224
2	CDK2	26	96.681	0.09664694
3	MYC	24	184.4955	0.09607843
4	CDK4	24	105.04828	0.09626719
5	CDK1	23	91.59032	0.09607843
6	CCND1	21	52.78467	0.09551657
7	CCNB1	21	36.13899	0.09514563
8	MDM2	20	68.274475	0.09551657
9	CDKN2A	19	19.204012	0.0945946
10	CDKN1B	19	25.033052	0.09477756
11	RB1	17	13.2853775	0.09423077
12	CDK6	17	30.316168	0.09423077
13	CCNA1	17	18.685257	0.09386973
14	ATM	16	34.02262	0.0945946
15	CCNE1	15	7.5703015	0.09386973
16	EP300	15	34.708378	0.094412334
17	CCNB2	13	8.407959	0.0926276
18	HSP90AA1	13	243.90038	0.094412334
19	CDC25C	12	6.867624	0.09315589
20	CDKN2B	11	2.432634	0.09315589
21	CCND2	11	2.3213787	0.09280303
22	SFN	11	81.153076	0.092979126
23	CREBBP	10	8.687286	0.09280303
24	CKS1B	10	0.6835514	0.09210526
25	NPM1	9	5	0.093333334
26	CDC25B	9	1.7154195	0.09210526
27	CDKN2C	8	0.43030304	0.09227872
28	SIRT1	8	2.8873017	0.092979126
29	BUB1B	6	0.5644527	0.0917603
30	RPA1	6	0.1	0.09193246
31	USP7	5	1.0888889	0.09141791
32	NCL	5	0.22222222	0.09193246
33	DAXX	5	0.75	0.09090909
34	C3	4	7	0.02173913
35	CDC37	3	1.3333334	0.09090909
36	CFP	3	1	0.02172949
37	NR3C2	2	152	0.08844765
38	HSD11B2	2	78	0.08291032
39	MAX	2	0	0.08941606
40	CFHR1	2	0	0.021719858
41	CFD	2	0	0.021719858
42	TP53BP2	1	0	0.09023941
43	HPGD	1	0	0.07777778
44	CD46	1	0	0.021710236
45	XDH	1	0	0.020408163
46	BTN1A1	1	0	0.020408163
47	BIN1	1	0	0.08925319
48	ANPEP	1	0	0.08657244
49	MAOA	1	0	0.020408163
50	ADH1C	1	0	0.020408163

### CDKN2A is significantly associated with the prognosis of CRC

**Fig. 5:**
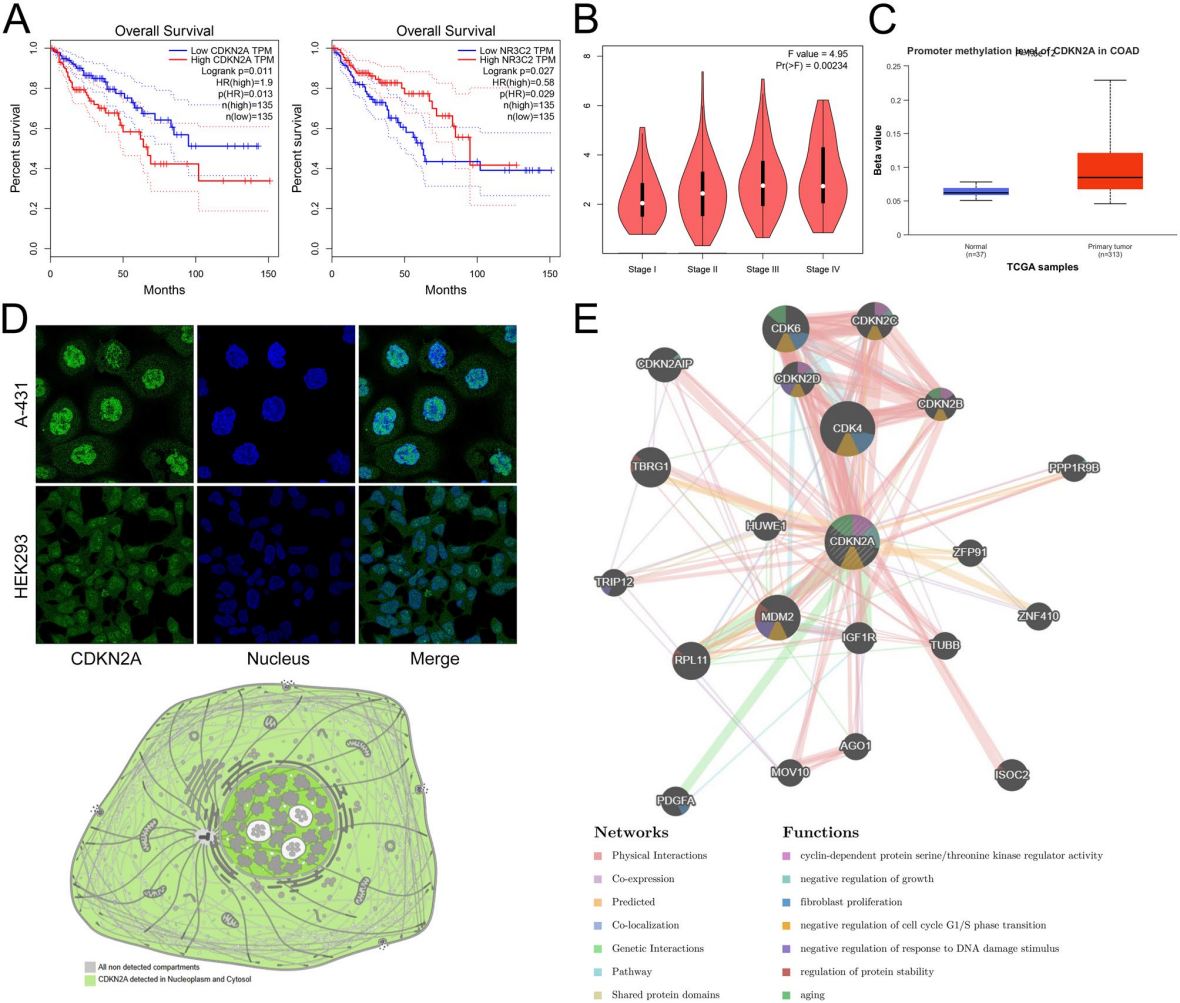
Analyses of CDKN2A expression characteristics, cellular localization and clinicopathological significance. (**A**) Relationship between CDKN2A expression and NR3C2 expression, and overall survival of CRC patients in GEPIA database. (**B**) The relationship between CDKN2A expression and clinical stage of CRC patients in GEPIA database. (**C**) CDKN2A promoter methylation levels in CRC patients were analyzed by UALCAN database. (**D**) HPA database was applied to the perform the analysis of CDKN2A subcellular localization. (**E**) GeneMANIA database was used to identify genes with similar functions to CDKN2A and a PPI network was constructed. The color of the nodes is related to protein function, while the color of the lines represents the type of protein interaction

GEPIA database was used to analyze the relationship between the expression of 14 genes and the survival and stage of patients. The results showed that high expression of CDKN2A was significantly correlated with shorter OS in CRC patients, while those with high expression of NR3C2 had longer OS (Fig. 5A, Figure S2). Additionally, CDKN2A expression was positively correlated with clinical stage (Fig. 5B, Figure S3). Previous studies have shown that CDKN2A promoter methylation plays a key role in cancer and is a potential biomarker for cancer prognosis [29, 30]. Interestingly, UALCAN showed that the CDKN2A promoter was hypermethylated in CRC cancer patients (Fig. 5C). Data from HPA database showed that CDKN2A was localized in the cytoplasm and nucleus of cells (Fig. 5D). With GeneMANIA database, 20 functionally similar genes of CDKN2A were obtained, and their functions focused on cell cycle, cell growth, and fibroblast proliferation (Fig. 5E).

### Results of molecular Docking

**Fig. 6:**
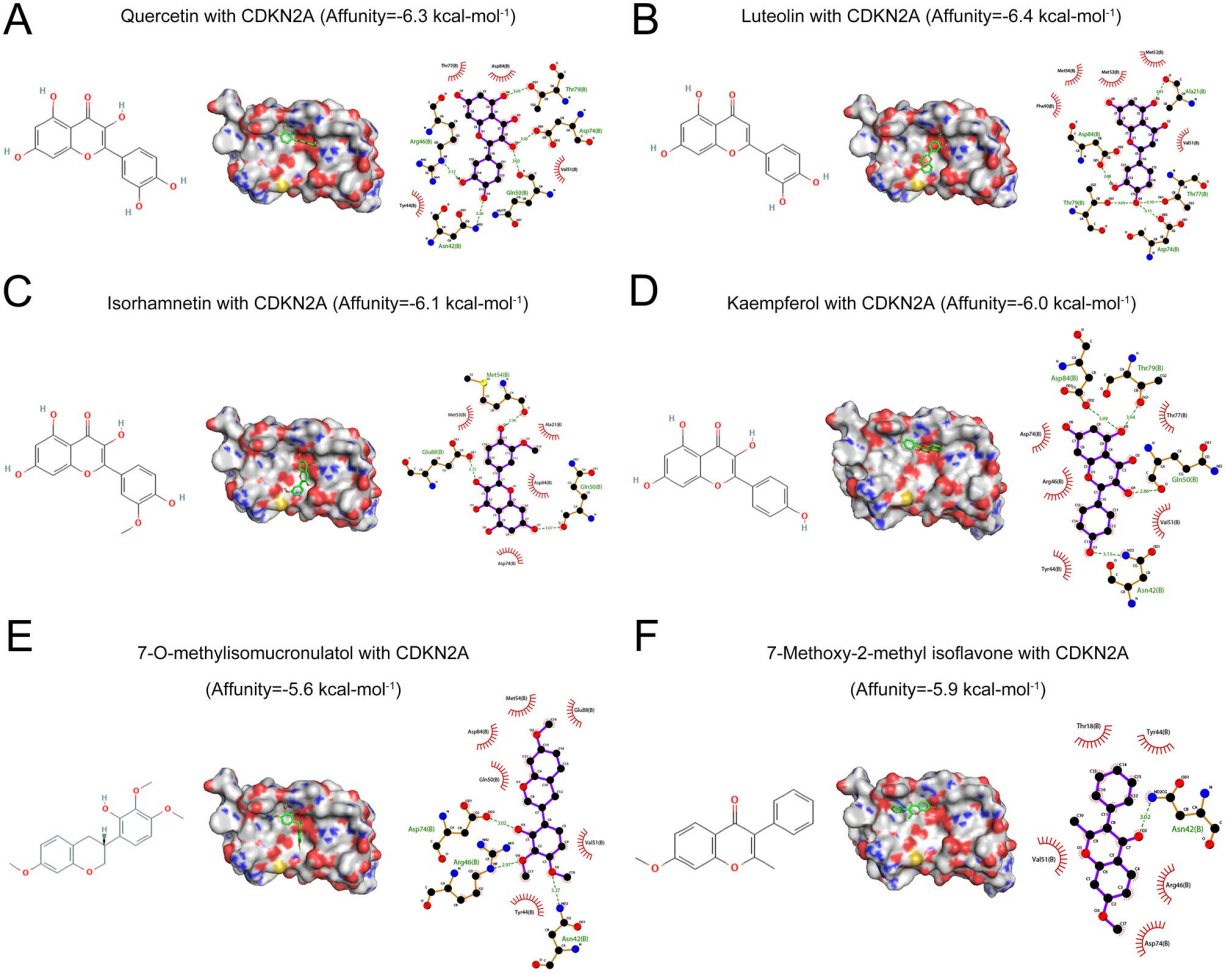
The results of molecular docking between CDKN2A and key bioactive components of CWQ. The binding pattern of quercetin (**A**), luteolin (**B**), isorhamnetin (**C**), kaempferol (**D**), 2-D structure of 7-O-methylisomucronulatol (**E**) and 7-Methoxy-2-methyl isoflavone (**F**) with CDKN2A

To explore the binding relationship between CDKN2A and the active ingredients of CWQ, the crystal structure of CDKN2A was searched from the PDB database (PDB: 1BI7), and the structure information of 6 active ingredients of CWQ was downloaded from PubChem database, including luteolin (MOL000006), kaempferol (MOL000422), quercetin (MOL000098), isorhamnetin (MOL000354), 7-O-methylisomucronulatol (MOL000378) and 7-Methoxy-2-methyl isoflavone (MOL003896). The results of molecular docking showed that quercetin mainly formed five hydrogen bonds with residues Arg46, Asn42, Gln50, Asp74 and Thr79 on CDKN2A protein (Fig. 6A). Luteolin mainly formed five hydrogen bonds with Ala21, Asp84, Thr79, Asp74, and Thr77 residues on CDKN2A protein (Fig. 6B). Isorhamnetin formed three hydrogen bonds with residues Met54, Glu88, and Gln50 on CDKN2A protein (Fig. 6C). Kaempferol formed four hydrogen bonds with Asp84, Thr79, Gln50, and Asn42 residues on CDKN2A protein (Fig. 6D). 7-O-methylisomucronulatol formed three hydrogen bonds with Asp74, Arg46, and Asn42 residues on CDKN2A proteins (Fig. 6E). 7-Methoxy-2-methyl isoflavone formed a hydrogen bond with Asp42 residues on CDKN2A protein (Fig. 6F).

### Quercetin inhibits CRC cell viability and induces G0/G1 cell cycle arrest

**Fig. 7:**
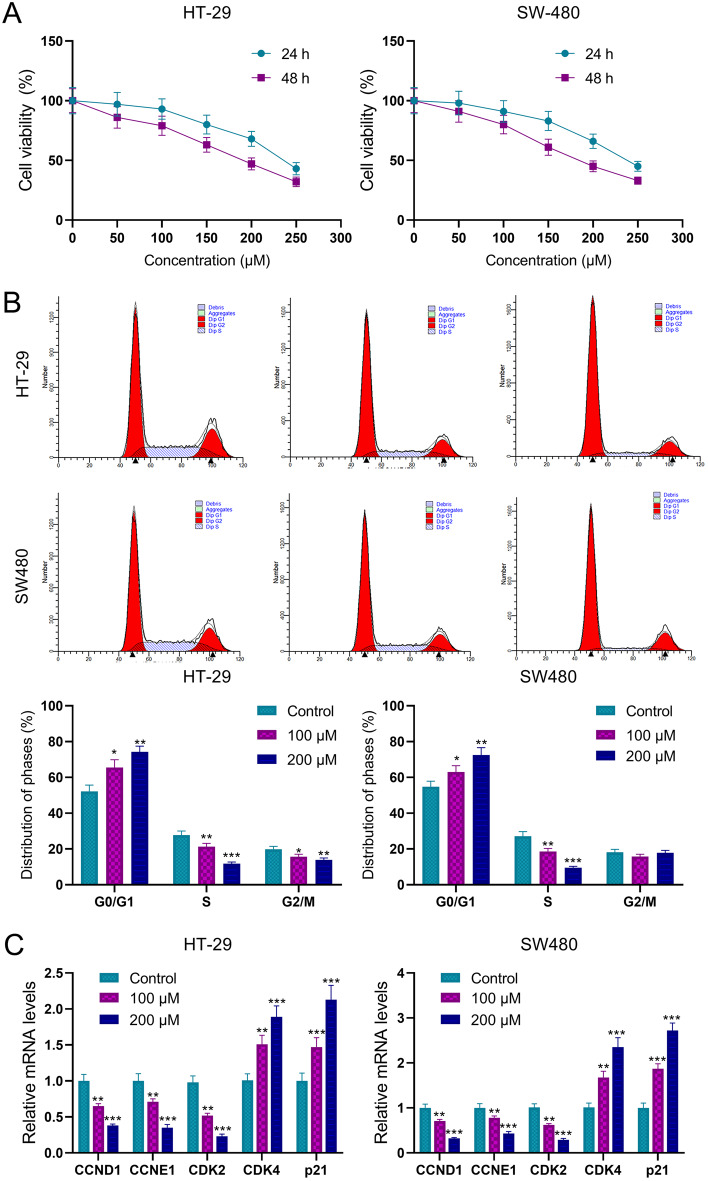
Quercetin inhibits CRC cell proliferation and induces G0/G1 cell arrest. (**A**) The viability of HT-29 and SW480 cells after quercetin treatment was detected by CCK-8 assay. (**B**) The effects of quercetin treatment on the cell cycle of HT-29 and SW480 were detected by flow cytometry. (**C**) mRNA expression levels of CCND1, CCNE1, CDK2, CDK4 and P21 were detected by qRT-PCR assay after the cells were treated with quercetin. All of the experiments were performed in triplicate. *P < 0.05, **P < 0.01, and ***P < 0.001 vs. control group

To further investigate the tumor-suppressive role of CWQ on CRC cells, the effect of different concentrations of quercetin, one of the main components of CWQ, on CRC cell proliferation was examined by CCK-8 assay. The results showed that quercetin inhibited CRC cell proliferation in a time - and dose-dependent manner. In HT-29 cells, the IC_50_ of quercetin at 24 h and 48 h was 237.1 and 184.8 µM, respectively. In SW480 cells, the IC_50_ of quercetin at 24 h and 48 h was 239.6 and 182.7 µM, respectively (Fig. 7A). Flow cytometry was then conducted, and the results showed that after quercetin treatment, the proportion of cells in G0/G1 phase was significantly increased, while the proportion of cells in G2/M and S phase was significantly decreased (Fig. 7B). The results of qRT-PCR showed that the expressions of CCND1, cyclin E1 and CDK2 were significantly decreased in the quercetin treatment group, while the expression levels of CDK4 and p21 were increased (Fig. 7C). These data implied that CWQ may suppress the proliferation and viability of CRC cells via inducing G0/G1 cell cycle arrest.

## Discussion

At present, the main treatment methods for CRC include surgery, targeted therapy, radiotherapy and chemotherapy [31]. Chemotherapy is commonly administered after surgery as an adjunct treatment for patients with advanced colorectal cancer [32, 33]. However, although chemotherapy drugs have certain anti-tumor effects, they can also cause serious adverse reactions. A variety of natural ingredients in traditional Chinese medicine have the characteristics of anti-tumor and low toxicity [34].

In this study, a total of 70 active ingredients and 823 targets of CWQ were identified. The main active ingredient of CWQ was identified to be quercetin, and the other potentially effective ingredients included luteolin, kaempferol, 7-Methoxy-2-methyl isoflavone, 7-O-methylisomucronulatol, and isorhamnetin. Quercetin is a plant flavonoid polyphenol found in a wide variety of fruits, vegetables and grains. Quercetin exerts its anti-cancer ability by regulating cyclins, pro-apoptosis, PI3K/Akt and mitogen-activated protein kinase (MAPK) pathways to reduce proliferation, induce apoptosis, cause cell cycle arrest and inhibit the mitosis process [35]. In this study, we used different concentrations of quercetin to treat HT-29 and SW-480 cells to analyze its effects. The results showed that quercetin inhibited the cell viability of HT-29 and SW-480 cells in a dose-dependent manner regardless of treatment for 24–48 h. These results indicate that CWQ has the potential to inhibit the growth of cancer cells. Luteolin shows the potential to treat diseases such as inflammatory diseases, hypertension and cancer, and the anti-inflammatory and antioxidant effects are its possible mechanisms for the treatment of CRC [36–40]. Kaempferol is a naturally derived flavonol that exerts its anticancer effects by inhibiting angiogenesis, stimulating apoptosis and inducing cell cycle arrest [41]. 7-Methoxy-2-methyl isoflavone is the main active ingredient of a variety of traditional Chinese medicine prescriptions including Yinchensini decoction and Fuzi Lizhong Tang, which plays an important role in treating various cancers such as gastric cancer and ovarian cancer [42–44]. 7-O-methylisomucronulatol is the main active ingredient of *Hedysarum Multijugum Maxim.*, which potentially effective in cancer treatment [42, 45]. Isorhamnetin is a flavonoid compound that has been found to affect the progression of multiple cancers including lung cancer [46], gastric cancer [47], breast cancer [48], CRC [49] and gallbladder cancer [50]. Additionally, enrichment analysis suggested that the targets of the ingredients in CWQ were associated with multiple biological processes and cancer-related pathways, including protein kinase activity, inflammatory response, AGE-RAGE signaling pathway, cellular senescence, MAPK pathway, TNF pathway and HIF-1 pathway. Taken together, in CRC treatment, CWQ exhibits tumor-suppressive properties through multiple bioactive components and downstream pathways.

To further identify the pivotal target of CWQ, in this study, based on TCGA data, DEGs and WGCNA were used for further investigation, and 6356 and 1901 CRC-related genes were obtained, respectively. 2980 CRC-related genes were obtained from DisgeNET, GeneCards, MalaCards, OMIM and CTD disease databases. After intersecting these targets, 14 key genes/targets were identified. Among these genes, it was revealed that CDKN2A was the only candidate, which was highly expressed in CRC tissues, and meanwhile significantly associated with short OS and higher clinical stage of CRC patients. Importantly, molecular docking showed that the main bioactive components of CWQ had good binding affinity with CDKN2A, and the binding affinities were lower than − 5.5 kcal/mol. The binding ability of CDKN2A to luteolin was the lowest (affinity=-6.4 kcal/mol), followed by quercetin (affinity=-6.3 kcal/mol). These data suggest that CDKN2A was the most important target of CWQ in CRC treatment. A lot of studies based on network pharmacology fail to identify the crucial targets of a compound preparation or a herbal medicine formula due to the large number of obtained targets from multiple ingredients. Our study suggests that additional bioinformatic analyses are helpful for noise reduction, and identifying the crucial targets which were indeed involved in disease progression and druggable.

In addition, CDKN2A promoter methylation is a common epigenetic event and an important factor leading to cell proliferation and uncontrolled tumor progression. CDKN2A hypermethylation is closely associated with poor prognosis and increased macrophage infiltration of CRC patients [51–53]. The gene of CDKN2A is located in chromosome 9p21, encoding the proteins p14^ARF^ and p16^INK4a^. p14^ARF^ can activate and stabilize p53 pathway to participate in the G1 and G2/M phase arrest of cancer cells [54], while p16^INK4a^ blocks the progression of G1/S cell cycle by preventing the phosphorylation of Rb [55, 56]. According to the functional analysis of CDKN2A and its interacting genes, CDKN2A can regulate biological processes such as cell growth, cell cycle and cell aging, which is consistent with previous results [54–56]. Interestingly, even though hypermethylation usually leads to low expression of the genes, CDKN2A is reported to be high expressed in CRC tissues, and its high expression implies poor prognosis of the patients [57, 58]. These reports suggest that the dysregulation of CDKN2A in CRC cells is resulted from posttranscriptional modification, and further studies are needed to elucidate the specific molecular mechanisms involved.

Considering in-silico data suggested CWQ regulated multiple targets and pathways involved in cell cycle progression, quercetin, the most important active component of CWQ, was selected for in vitro experiments, to investigate its role on modulating cell proliferation and cell cycle progression. The results showed that quercetin could cause G0/G arrest, inhibit the expression of CCND1, CCNE1 and CDK2, and promote the expression of CDK4 and p21. We suppose that quercetin may promote the entry of G1 phase by regulating the expression of CDK4 [59]. On the other hand, quercetin inhibits the activation of cyclinE-CDK2 complex by promoting the expression of p21, preventing the cell cycle from entering S and G2/M phases from G1 phase [60, 61], resulting in cell cycle arrest in G0/G1 phase.

There are some limitations to the study. First, the biological functions of other components of CWQ on CRC cells were not explored. Secondly, this study did not investigate the effects of CWQ on the migration, invasion and chemotherapy sensitivity of CRC cells, which are crucial procedures during disease progression. Additionally, the findings in the present work requires further validation in animal models and preclinical models (such as organoid) [62], which will provide more convincing evidence to support the clinical application of CWQ. These topics deserve further investigation in the following work.

## Conclusion

**Fig. 8:**
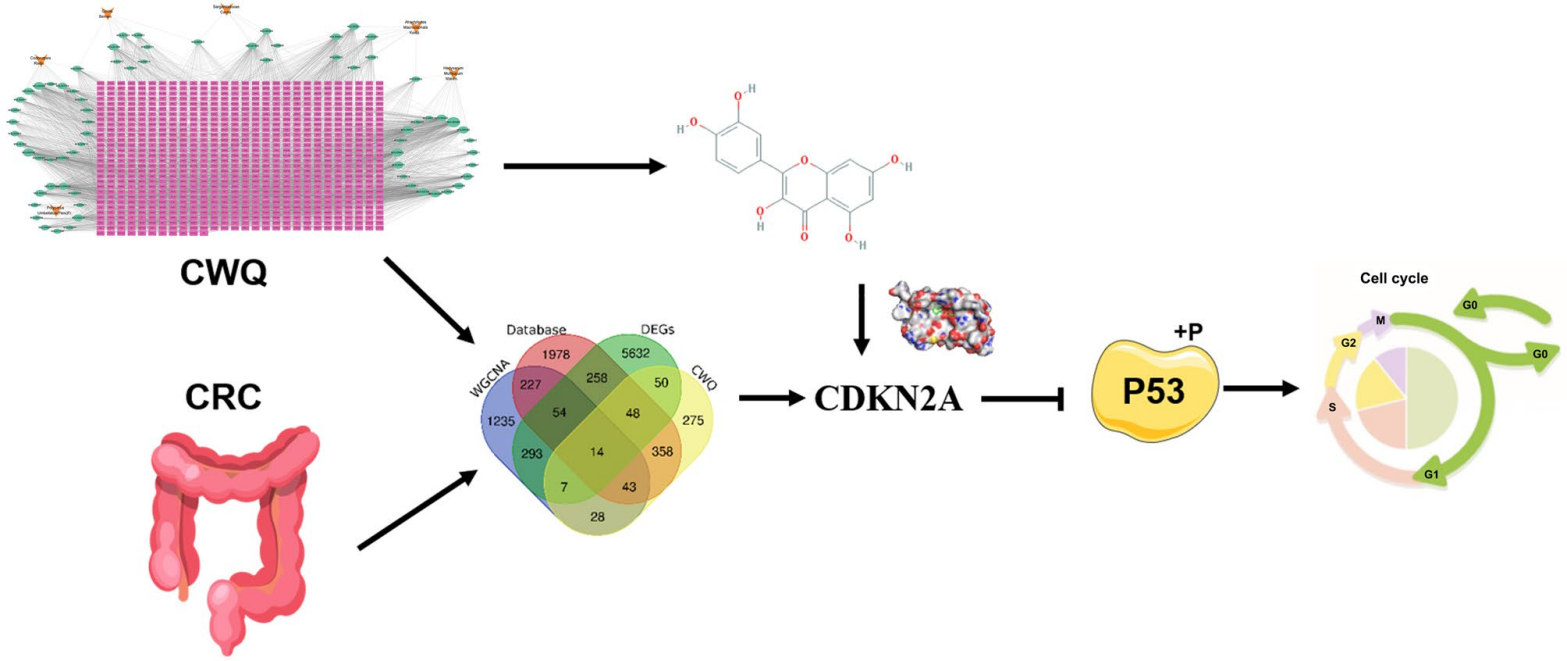
Graphic Abstract: Combination of WGCNA, network pharmacology and molecular docking identifies CDKN2A as a crucial target of CWQ in CRC treatment

The combination of WGCNA, network pharmacology, and molecular docking identifies CDKN2A as a crucial target of CWQ in CRC treatment (Fig. 8). CWQ may regulate the aggressiveness of CRC cells via modulating cell cycle-related proteins. This study provides a theoretical basis for the clinical application of CWQ in CRC treatment.

## Electronic supplementary material

Below is the link to the electronic supplementary material.

Supplementary Material 1

Supplementary Material 2

## Data Availability

The data and materials is available from the corresponding author via e-mail if the request is reasonable.
