# Supplementary material for: Network pharmacological approach combined with weighted gene co-expression network analysis identifies CDKN2A as the keg target of Changweiqing against colorectal cancer
Source: Hereditas. 2025 Mar 10;162:33. doi: 10.1186/s41065-025-00405-8 (PMC11892207; doi:10.1186/s41065-025-00405-8)
Supplement: Supplementary file 2 — Supplementary Material 2 [file 41065_2025_405_MOESM2_ESM.docx]

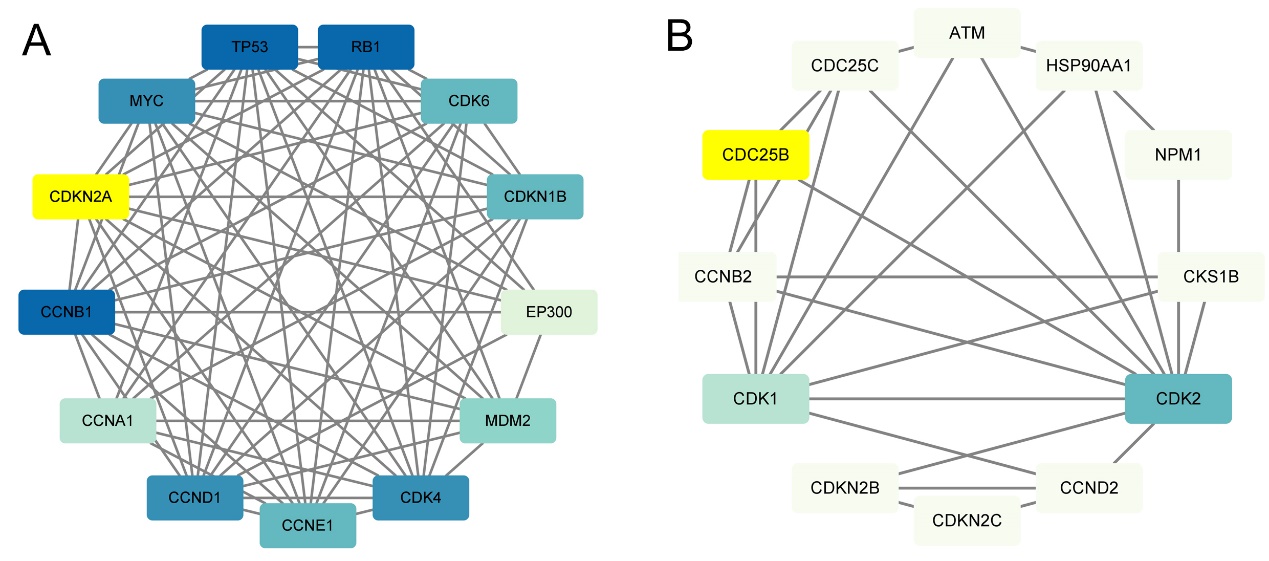


Table S1. MCODE plug-in in cytoscape software was used to classify the 54 genes into module 1 (A) and module 2 (B), and the yellow nodes indicated the genes among the 14 key targets.


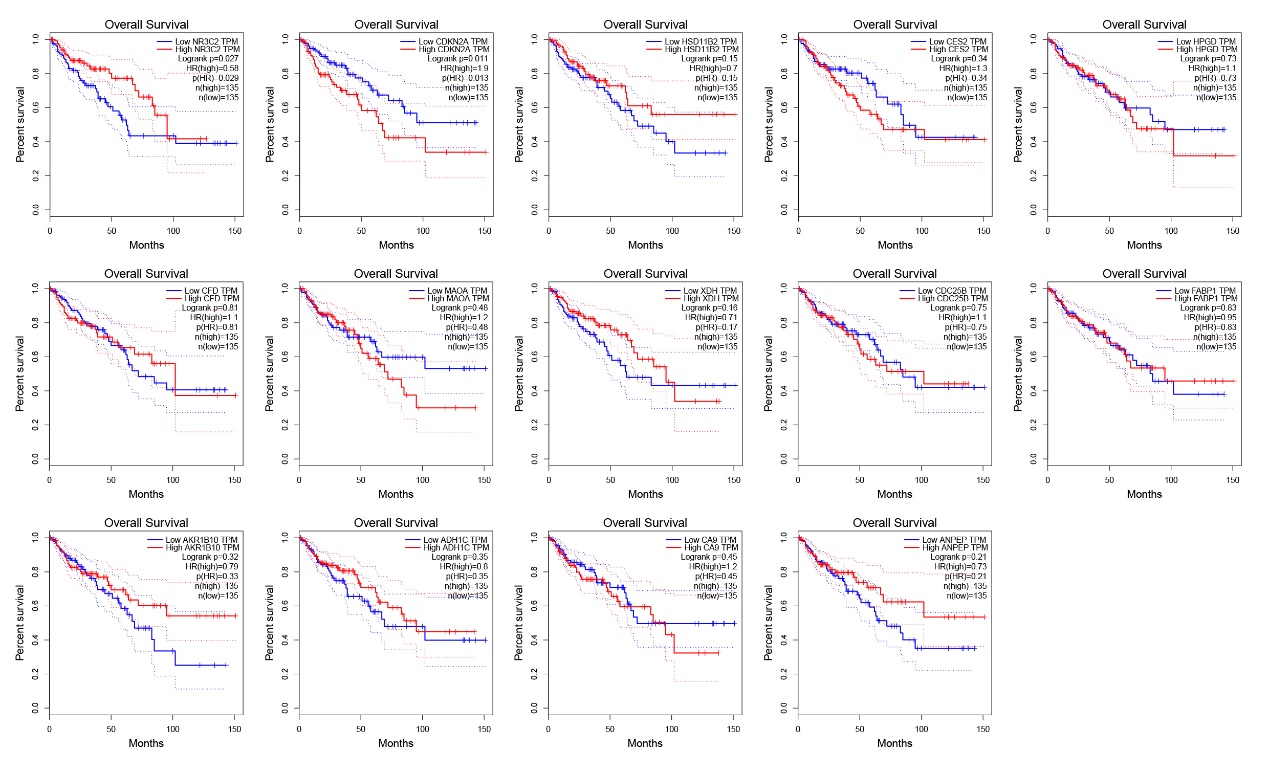


Table S2. GEPIA database was applied to analyze the relationship between the 14 genes and the overall survival times of CRC patients.


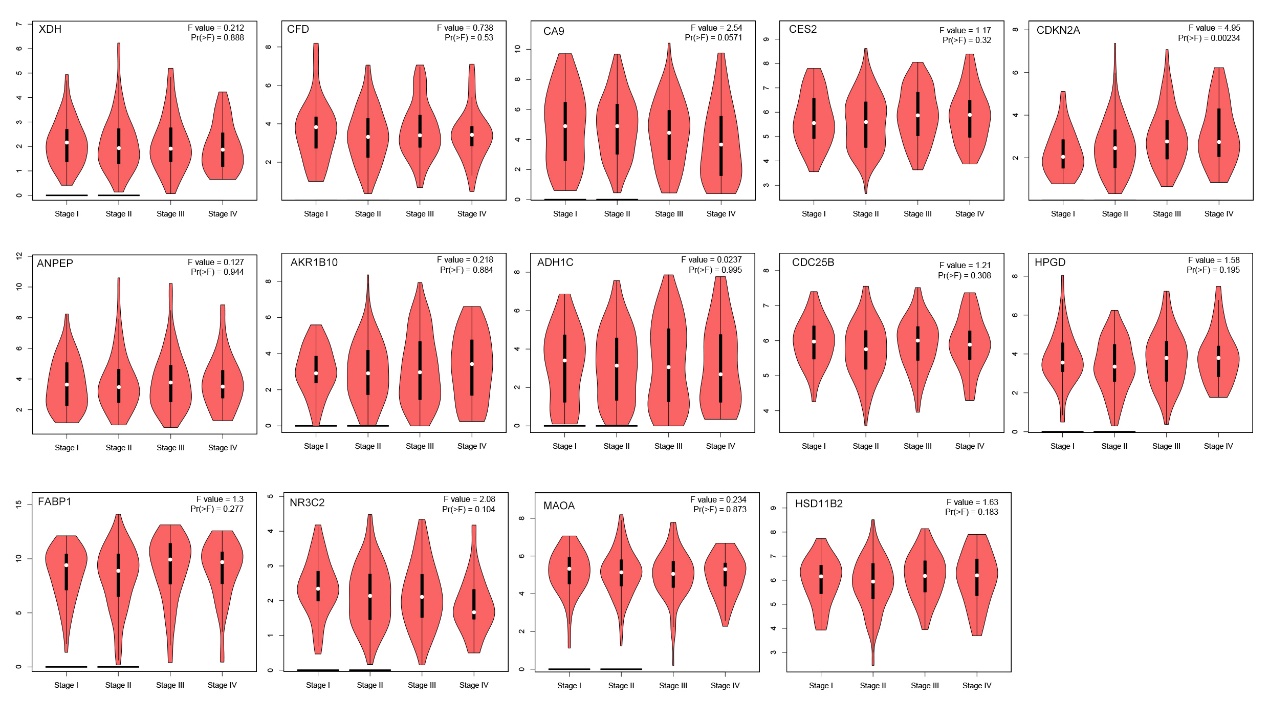


Table S3. GEPIA database was applied to analyze the relationship between the 14 genes with the clinical stage of CRC patients.
